# Genetic surgery for a cystic fibrosis-causing splicing mutation

**DOI:** 10.1016/j.omtm.2023.101177

**Published:** 2024-01-02

**Authors:** Mattijs Bulcaen, Marianne S. Carlon

**Affiliations:** 1 Laboratory for Molecular Virology and Gene Therapy, Department of Pharmaceutical and Pharmacological Sciences, KU Leuven, Leuven, Belgium; 2 Center for Molecular Medicine, KU Leuven, Leuven, Belgium; 3 Laboratory of Respiratory Diseases and Thoracic Surgery (BREATHE), Department of Chronic Diseases and Metabolism, KU Leuven, Leuven, Belgium

Cystic fibrosis (CF) is one of the most common inherited life-threatening diseases worldwide and is caused by mutations in the *CFTR* gene that lead to absent or aberrant CFTR protein. CFTR is an ion channel essential for fluid homeostasis over epithelial membranes, and disruption of its function leads severe clinical symptoms in multiple organs. For a subset of mutations, including the most prevalent F508del, small-molecule CFTR modulators have been developed and have revolutionized CF therapy. However, people with CF (pwCF) carrying severe missense, splicing, or nonsense mutations are, based on their genotype, not eligible for these highly effective modulator therapies (HEMTs), urging the investigation of genetic medicines.

Walker et al. describe the molecular equivalent of precision surgery.^1^ In their recent study, the authors leverage a cutting SpCas9 and two single guide RNAs (sgRNAs) to snip open the genome and remove a cryptic splicing signal (c.3718-2477C>T, legacy name 3849+10kbC>T) allowing them to bring the *CFTR* mRNA back to its normal sequence and length. To make the cut quick and free from undesired complications, the authors leverage the transient nature of lipid nanoparticles decorated with airway-targeting peptides for the delivery of their surgical tools.

With an allelic frequency of 0.815% (www.cftr2.org), c.3718-2477C>T ranks 10^th^ in the list of CF-causing mutations. The C>T transition dictates the incorporation of a pseudo-exon containing a premature stop codon, leading to a truncated and dysfunctional CFTR protein. In 2017, Sanz et al. demonstrated in a HEK293T model that the cryptic signal can be precisely excised using CRISPR-Cas9 and two sgRNAs.^2^ SpCas9, an RNA-guided DNA endonuclease initially discovered in *Streptococcus pyogenes*, can be considered a biochemical surgeon’s favorite tool: it is fast, predictable, and reasonably safe to operate. Sanz and colleagues showed that the removal of a 178 bp region in intron 22 that contains c.3718-2477C>T, allowed to restore CFTR splicing in a minigene model.^2^

These results provided convincing proof of concept, but further validation in more relevant models was warranted. More translational cell models that are frequently used in the CF field include FRT, 16HBE or CFBE airway epithelial cell lines, patient-derived intestinal organoids, patient-derived human nasal epithelial (HNE) cells, or human bronchial epithelial (HBE) cells.^3^

In a joint effort, researchers from University College London and University of Cork delivered SpCas9 and the two sgRNAs to BMI1-transduced CFBE3849 CFBE cells homozygous for c.3718-2477C>T.^1^ CFBE cells can be differentiated at air-liquid interface, leading to endogenous CFTR expression that can be functionally assessed by Ussing chamber electrophysiology. To deliver the gene-editing components, SpCas9-sgRNA ribonucleoproteins (RNPs) were encapsulated in DOTMA-DOPE lipid nanoparticles (LNPs) using cationic peptides for efficient RNP loading and functionalized with a cyclic peptide earlier identified for the efficient targeting of airway cells.^4^

In their study, Walker et al. show that LNP-mediated delivery to CFBE cells led to high levels of gene editing (>60%) and did not impair their differentiation capacity.^1^ Moreover, gene editing led to an increase of correctly spliced *CFTR* mRNA (increase from 25% to 66% wild-type [WT] CFTR transcripts) and translated to a significant increase in CFTR function measured through electrophysiology (from 3.3 to 5.7 μA/cm^2^). The current measured in the gene-edited cells was roughly 25% of that measured in WT cells; if achieved *in vivo*, these levels of functional correction are expected to deliver significant clinical benefit.^5^

To assess the safety of the genetic surgery, the authors performed Sanger sequencing of these regions, revealing no off-target scarring, although the authors remark that for clinical application, a much more thorough and sensitive safety analysis will be required.^1^

The current work fits in an ongoing effort of the CF field to answer the high unmet medical need of the so-called “last 10%,” i.e., pwCF that are, based on their specific genotype, ineligible for HEMTs and reside in a pre-modulator era. Aside from severe missense mutations, these genotypes consist of splicing, insertion or deletion (indel), or nonsense mutations that are mechanistically not expected to respond to any of the current or future modulator therapies. To tackle this unmet medical need, the CF field works hard on gene addition as well as gene-editing approaches (see Table 1). Indeed, since the clinical approval of HEMTs for the most common mutation, F508del, as well as for gating and residual function mutations, drug-refractory mutations have been high on the research agenda (see Table 1). c.3718-2477C>T is a residual function mutation, and pwCF carrying such a mutation are now eligible for the approved CFTR modulators in the USA (https://www.fda.gov/).^6^ In Europe, only pwCF carrying the mutation in combination with an F508del allele are eligible for Symkevi (tezacaftor/ivacaftor) and Kaftrio (iva-/teza-/elexacaftor) (https://www.ema.europa.eu/), implying that still not every pwCF carrying this mutation is sure of a causative therapy to date.

**Table 1:** Overview of gene-editing strategies published for correction of cystic fibrosis causing CFTR mutations

Rank in CFTR2	Legacy name	cDNA name	Allele frequency (%)	Modulator eligible	Strategy	References
#1	F508del	c.1521_1523delCTT	69.74	FDA and EMA	• HDR (zinc finger nuclease + template) • HDR (SpCas9 + template) • HDR (TALEN + template) • HDR (zinc finger nuclease + template) • HDR (SpCas9 + template) • HDR (TALEN + template) • HDR (SpCas9 + template) • HDR (SpCas9 + template) • HDR (TALEN + template) • HDR (SpCas9 + template) • HDR (SpCas9 + template) • prime editing (PE3) • HDR (SpCas9 + template)	Lee et al., Biores Open Access., 2012 Schwank et al., Cell Stem Cell, 2013 Sargent et al., Methods Mol Biol., 2014 Crane et al., Stem Cell Reports, 2015 Firth et al., Cell Rep., 2015 Suzuki et al., Mol Ther Nuceic Acids., 2016 Hollywood et al., Sci Rep., 2016 Ruan et al., Mol. Ther. Nucleic Acids, 2019 Fleischer et al., Mol. Ther., 2020 Suzuki et al., Mol Ther, 2020 Vaidyanathan et al., Cell Stem Cell, 2020 Geurts et al., Life Sci Alliance., 2021 Wei et al., Nat Commun., 2023
#2	G542X	c.1624G>T	2.54	–	HDR (SpCas9 + template)	Wei et al., Nat Commun., 2023
#3	G551D	c.1652G>A	2.1	FDA and EMA	–	–
#4	N1303K	c.3909C>G	1.58	–	–	–
#5	R117H	c.350G>A	1.31	FDA and EMA	–	–
#6	W1282X	c.3846G>A	1.22	–	• adenine base editing (xCas9-ABEmax) • adenine base editing (SpCas9-ABEmax) • adenine base editing (NG-ABEmax) • homology-directed repair (2× nCas9 + template) • homology-directed repair (SpCas9 or SaCas12a) • adenine base editing • prime editing	Geurts et al., Cell Stem Cell, 2020 Jiang et al., Nat Commun., 2020 Krishnamurthy et al., Nuc Acids Res., 2021 Suzuki et al., Fron Genome Ed., 2022 Santos et al., J Cyst Fibros., 2022 Mention et al., Hum Mol Genet., 2023 Li et al., PLoS One, 2023
#7	R553X	c.1657C>T	0.93	–	• adenine base editing (xCas9-ABEmax) • adenine base editing (SpCas9-ABEmax)	Geurts et al., Cell Stem Cell, 2020 Krishnamurthy et al., Nuc Acids Res., 2021
#8	621+1G>T	c.489+1G>T	0.93	–	–	–
#9	1717-1G>A	c.1585-1G>A	0.86	–	–	–
#10	3849+10kbC>T	c.3718-2477C>T	0.82	FDA	• excision of cryptic splicing signal (SpCas9 + 2× sgRNA) • allele-specific excision of splicing signal (AsCas12a) • adenine base editing (NG-ABEmax) • *excision of cryptic splicing signal (SpCas9 + 2× sgRNA)*	Sanz et al., PLoS One, 2017 Maule et al., Nat comm, 2019 Krishnamurthy et al., Nuc Acids Res, 2021 Walker et al., *Mol Ther Methods Clin Dev., 2023*
#11	2789+5G>A	c.2657+5G>A	0.72	–	• adenine base editing using NG-ABEmax	Amistadi et al., Mol Ther., 2023
#13	R1162X	c.3484C>T	0.46	–	• adenine base editing (xCas9-ABEmax)	Geurts et al., Cell Stem Cell, 2020
#22	3272-26A>G	c.3140-26A>G	0.33	FDA	• allele-specific excision of splicing signal (AsCas12a)	Maule et al., Nat comm, 2019
#130	R785X	c.2353C>T	0.02	–	• adenine base editing (xCas9-ABEmax)	Geurts et al., Cell Stem Cell, 2020

Abbreviations HDR: homology-directed repair, FDA: U.S. Food and Drug Administration, EMA: European Medicines Agency. Gene editing approaches are listed only; mutation-agnostic approaches such as gene addition or super-exon strategies are excluded but have been reviewed elsewhere.^10^

The SpCas9-mediated double-strand break excision strategy used in this study effectively rescues the splicing defect caused by the c.3718-2477C>T, also confirmatory shown by others (Table 1; Maule et al.^7^).

Many of these approaches leveraged cleaving Cas endonucleases, which are often superior in efficiency compared to other gene editing technologies, but also come with significant side effects under the form of p53-induced genotoxicity and chromosomal rearrangements.^8^ Non-homologous end-joining (NHEJ)-based strategies have the advantage of working in non-dividing cell types but can also be considered a blunt knife because they deliver a mixture of indels at the cut site. It should be remarked that this side damage can be tolerated when cutting in intronic sequences but not in CFTR-coding exons. To operate these, more precise tools like base editing, prime editing, or homology-dependent repair (HDR) are required. Some of these have already been applied for correction of the c.3718-2477C>T too (Table 1).

For gene-editing purposes, being transient is key. Just as for non-molecular surgery, it is important to be in and out as fast as possible and to leave no traces except for the intended intervention. Of the delivery modalities that are available today, RNP-based LNPs belong to the fastest and most transient vehicles to shuttle in gene-editing cargo. The nanoparticles described in this study provide SpCas9 precomplexed with its sgRNAs as a ready-to-go molecular machine.^1^ They do not contain any mRNA or DNA that can amplify the dose or prolong the duration of exposure. In recognition of their low immunogenicity and standardizable production, several groups besides the authors of this work reported efforts to develop airway-tropic LNPs.^9^

In conclusion, the study from Walker and colleagues provides nice groundwork on the ability to permanently remove a deep intronic splicing mutation in the *CFTR* gene using CRISPR-Cas9 mediated excision.^1^ They showed convincingly that delivery through lung-targeted nanoparticles effectively restored CFTR splicing and function in relevant patient-derived primary airway cell models, underscoring their translational potential. The next steps likely will involve the further testing of delivery and editing in the presence of the well-known and non-trivial barriers that pose a challenge to efficiently reaching sufficient numbers of cells in an intact airway epithelium lined with viscous mucus. In that light, both systemic and apical targeting of the airway epithelium are active areas of investigation.
